# Energy Deregulation Precedes Alteration in Heart Energy Balance in Young Spontaneously Hypertensive Rats: A Non Invasive *In Vivo* ^31^P-MR Spectroscopy Follow-Up Study

**DOI:** 10.1371/journal.pone.0162677

**Published:** 2016-09-13

**Authors:** Veronique Deschodt-Arsac, Laurent Arsac, Julie Magat, Jerome Naulin, Bruno Quesson, Pierre Dos Santos

**Affiliations:** 1 L'Institut de Rythmologie et Modélisation Cardiaque LIRYC, Université de Bordeaux, Pessac, France; Inserm U1045 CRCTB, Université de Bordeaux, Bordeaux, France; 2 L'Institut de Rythmologie et Modélisation Cardiaque LIRYC, Université de Bordeaux, Pessac, France; Inserm U1045 CRCTB, Université de Bordeaux, Bordeaux, France; Hôpital cardiologique Haut-Lévêque, CHU de Bordeaux, Pessac, France; Temple University, UNITED STATES

## Abstract

**Introduction:**

Gradual alterations in cardiac energy balance, as assessed by the myocardial PCr/ATP-ratio, are frequently associated with the development of cardiac disease. Despite great interest for the follow-up of myocardial PCr and ATP content, cardiac MR-spectroscopy in rat models *in vivo* is challenged by sensitivity issues and cross-contamination from other organs.

**Methods:**

Here we combined MR-Imaging and MR-Spectroscopy (Bruker BioSpec 9.4T) to follow-up for the first time *in vivo* the cardiac energy balance in the SHR, a genetic rat model of cardiac hypertrophy known to develop early disturbances in cytosolic calcium dynamics.

**Results:**

We obtained consistent ^31^P-spectra with high signal/noise ratio from the left ventricle *in vivo* by using a double-tuned (^31^P/^1^H) surface coil. Reasonable acquisition time (<3.2min) allowed assessing the PCr/ATP-ratio comparatively in SHR and age-matched control rats (WKY): i) weekly from 12 to 21 weeks of age; ii) in response to a bolus injection of the ß-adrenoreceptor agonist isoproterenol at age 21 weeks.

**Discussion:**

Along weeks, the cardiac PCr/ATP-ratio was highly reproducible, steady and similar (2.35±0.06) in SHR and WKY, in spite of detectable ventricular hypertrophy in SHR.

At the age 21 weeks, PCr/ATP dropped more markedly (-17.1%±0.8% vs. -3,5%±1.4%, *P*<0.001) after isoproterenol injection in SHR and recovered slowly thereafter (time constant 21.2min vs. 6.6min, *P*<0.05) despite similar profiles of tachycardia among rats.

**Conclusion:**

The exacerbated PCr/ATP drop under ß-adrenergic stimulation indicates a defect in cardiac energy regulation possibly due to calcium-mediated abnormalities in the SHR heart. Of note, defects in energy regulation were present before detectable abnormalities in cardiac energy balance at rest.

## Introduction

A number of experimental and clinical data suggest that alterations in cardiac energy balance may contribute to the various pathological features of the heart [1–3]. Energetic alterations might not simply be secondary to ventricular remodeling, but rather be an early feature in the course of cardiac disease [3]. Yet, limited evidence is available on the time course of progressive disruption in myocardial energy balance, which suggests that a better understanding could be gained from follow-up studies of heart energetics as heart disease develops.

Magnetic Resonance (MR) spectroscopy has become a relevant tool to assess myocardium energetics and has the potential for non-invasive investigations. Despite great interest in non-invasive MR techniques for follow-up studies, myocardial phosphocreatine (PCr) and adenosine triphosphate (ATP) levels are hardly detectable by MR spectroscopy *in vivo* due to sensitivity issues and cross-contamination from surrounding tissues. These difficulties culminate in rodent models due to reduced cardiac volume as a source of poor signal-to-noise ratio. Thus, the critical role assumed by rodent models in MR research on cardiac energy balance has been mostly based on isolated perfused hearts.

Energy balance in the mammalian heart refers to the dynamic homeostasis of ATP, PCr and related forms of biochemical potentials (mainly the phosphorylation potential ΔGp) within the myocardium. This homeostatic regulation dynamically matches cardiac energy demand and cardiac energy supply. The creatine kinase (CK) reaction serves as the prime cardiac energy reservoir, quickly, reversibly and permanently converting adenosine diphosphate (ADP) and PCr to ATP and creatine [4–6]. ^31^P MR spectroscopy allows quantifying myocardial CK metabolites PCr and ATP. While the level of ATP is strictly preserved thanks to the biomechanical characteristics of the CK reaction, any defect in the tissue energy status will affect the PCr level and therefore PCr/ATP ratio [7]. While the PCr/ATP ratio amounts to 4–5 in skeletal muscle at rest and 1.8–2.5 in heart of healthy mammals, the ratio classically drops to lower values in case of organ defects [8]. Calcium-acting hormones play a critical role in supply-demand matching as demonstrated in both theoretical and experimental ways [9–12]. The ß-adrenergic stimulation of the myocardium triggers important modulations in cardiac cell cytosolic calcium concentration and calcium transients, which addresses several targets in supply and demand parts of the energy metabolism in parallel (see *e*.*g*. [13]). Thanks to parallel activation of supply and demand rates [9,12], this calcium-mediated ß-stimulation gives rise to increased cardiac work with no drop in energetic intermediates PCr or ATP [14], which constitutes by definition a homeostatic regulation. As perturbations of cell calcium dynamics occur concomitantly with deteriorating cardiac energetics [15], this is a potential cause of progressive disruption in energy balance as heart disease develops.

Early perturbations in calcium dynamics inside cardiomyocytes are a characteristic of the young maturating spontaneous SHR [16,17], a genetic model of hypertension and ventricular hypertrophy [18,19]. Although the ultimate cause of myocardial dysfunction in the adult SHR is still unclear, a crucial role of the myocardial energy metabolism is suspected [20] as in many other cardiac diseases [21]. Data on cardiac energetics in the young SHR during the course of heart disease are scarce and hardly conclusive. Biochemical analyses of myocardial energy metabolites in excised SHR hearts lead to suspect a defect in the myocardial energy balance at rest arising around the age 20-weeks in a study where different rats were sacrificed at 5, 8, 10, 15, 20, 25 weeks of age [22]. No longitudinal study to date has explored myocardial energy fluctuations along weeks as the disease develops in the same animal, probably because of the technical challenge of obtaining non-invasive assessments of phosphorylated compounds in the rat heart *in vivo*.

Here we combined MR-Imaging and localized MR-Spectroscopy at high magnetic field (9.4T Bruker BioSpec) for assessing respectively the fraction of blood content ejected by the cardiac left ventricle during contraction (systole) and the energy status of the myocardic tissue in the left ventricle of control rats (WKY) comparatively with young rats who progressively develop spontaneous hypertension (SHR) during maturation. We obtained good compromise between high signal-to-noise (S/N) ratio and reasonable acquisition time (<3.2 min) when using a Outer Volume suppression (OVS) technique for spatial localization of the ^31^P NMR spectra on the heart. Myocardial PCr and ATP levels were assessed weekly in each animal between 12 and 21 weeks of age in order to evaluate the time course of energy balance as the disease develops. At the age 21 weeks, a bolus of isoproterenol (a ß-adrenoreceptors agonist) was intravenously injected into the tail during MRS measurements to evaluate the calcium-mediated ß-adrenergic regulation of energy balance thanks to the dynamic response of the myocardial PCr/ATP-ratio.

## Methods

### Ethical approval

All animal protocol were approved by the Committee on the Ethics of Animal Experiments of Bordeaux (CEEA50 permit number DIR1326) and performed in accordance with the Guide for the Care and Use of Laboratory Animals of the National Institutes of Health (Publication No. 85–23, revised 1996) in an authorized environment (PTIB A-33-318-2). Certified experimenters controlled all the procedures in order to minimize suffering and to early detect any indicator of pain, distress or suffering on the animals. All the rats were euthanized at the end of the experimental procedure using an IP injection of buffered and diluted barbiturates with local anesthetic.

### Animals

Male spontaneously hypertensive rats (n = 4) (SHR) and their age-matched Wistar-Kyoto (n = 4) (WKY) counterparts obtained from Janvier Laboratories (France) were involved in this follow-up, non-invasive, MR study. The animals were aged 10 weeks (body mass 343g ± 19) at arrival in the laboratory, 12 weeks at onset of experiments and 21 weeks at ultimate *in vivo* measurements. They were housed in environmentally controlled rooms with 12-hour light and dark cycles, fed a standard certified laboratory diet *ad libitum* and had permanent access to water. All experiments were conducted under the supervision of a certified experimenter, between 10 am and 1 pm, during the early absorptive (fed) state. After completing each non-invasive MRS examination, the anesthesia (flushed gas through a face mask) was stopped; the animals woke up within 5 min. They were placed back in the cage of the control room under the supervision of a certified experimenter in charge of detecting any sign of suffering, despite the absence of traumatic intervention in our in vivo experiments.

### ^1^H MR-Imaging and ^31^P MR-Spectroscopy set-up

All the MR procedures were performed in a horizontal 9.4T, 30 cm inner diameter bore system (Bruker BioSpin MRI, Ettlingen Germany) and used a tunable ^1^H (400.34 MHz) /^31^P (162.06 MHz) 20 mm surface coil for transmission and reception. The most adequate MR routine for *in vivo* cardiac spectroscopy in the rat was determined experimentally by using a phantom (Eppendorf tubes containing PCr and ATP) prior to *in vivo* experiments on animals. In every experiment, the coil was positioned at the magnet isocenter, and topped by either Eppendorf tubes for prior set up procedures or by the chest of the animal for *in vivo* experiments.

### Localized ^31^P MR spectra

The conventional ISIS (Image Selected In vivo Spectroscopy) method offers precise spatial localization but requires long acquisition times. Instead, we chose a standard non localized pulse-acquisition sequence preceded by 6 spatially selective saturation band surrounding the volume of interest (so called “outer volume suppression”- OVS). This method offers optimal signal to noise ratio that can be exploited to reduce the acquisition time for transient analysis of metabolic changes through quantification of the PCr/ATP ratio. Acquisition parameters were: 200 μsec block pulse, 90° flip angle, 1.5 s repetition time, 8 kHz bandwidth (BW), 4096 points, 64 averages. Spatial localization was achieved with 6 saturation bands positioned around the volume of interest. Each saturation band was composed of a hyperbolic secant pulse (15 kHz BW, 1.35 ms pulse duration) followed by spoiling gradients of 1.8 ms duration and 3mT/m amplitude. Since spatial selectivity efficiency may differ as a function of the peak location in frequency, a careful analysis of the saturation efficiency was conducted prior to in vivo spectroscopy. Spatial selectivity of the OVS method was quantified on two adjacent tubes containing 20 mM PCr only and 8 mM ATP only, respectively. The volume of interest included initially the two tubes; then a saturation slice was iteratively shifted by steps of 1mm so that it progressively covered up the PCr tube until maximal attenuation of the PCr signal. For purpose of S/N comparison, the ISIS method was ran with the following sequence parameters: 200 μsec block pulse, 90° flip angle, 64 averages (each average being a combination of 8 measurements with 1.5 s repetition time, see [23] for details), 8 kHz bandwidth, 4096 points. Each spectrum was processed identically (see below for details).

### Spectra analysis

The free induction decays were low-pass filtered by multiplication by an exponential decay function that generated a 10Hz line broadening after Fourier transformation. Quantification of the signals integrals for each peak was carried out using multiple lorentzian peaks fits and cubic baseline (IgorPro, WaveMetrics). Noise level was estimated by computing the standard deviation (σ_n_) of the spectrum in the bandwidth included between 7 and 10 ppm. The S/N was computed for each peak by dividing each signal integral derived from the fitting routine by σ_n_. Because of the difficulties involved when determining absolute quantities using *in vivo* spectroscopy, the PCr/ATPγ ratio was used as a surrogate of energy balance. Respective levels of myocardial PCr and ATP contents were corrected for partial saturation effects using a factor of 1.09 determined in separate *in vivo* experiments that included partially relaxed (TR = 3s, 5s, 10s and 15s) and fully (TR = 20s) relaxed acquisitions.

### MR procedures in vivo

MR spectra as well as cine MR images were obtained in rats in vivo maintained under general anesthesia by the inhalation of 2% isoflurane mixed with 80% air and 20% oxygen. The animal was positioned prone in a purpose-built temperature regulated cradle. ECG electrodes were attached into the forepaws and a respiration loop was placed against the thorax. Body temperature was continually monitored with a rectal probe. Respiration and ECG inputs were monitored continuously. ECG signal was used as trigger input for MRI and MRS at the same point in the cardiac cycle.

Several scout images were acquired to determine the short axis view of the rat heart. First, a shimming was performed with the use of the 1H coil to optimize the magnetic field homogeneity in the heart. This shimming procedure allowed to obtain a proton line width of approximately 50 Hz at the center of the heart. An ECG and respiratory trigged FLASH cine images were performed acquired in 3 slices (basal, medium and apical plans). The imaging parameters used were as follows: 60x60 mm field of view (FOV), 192x192 matrix size, 1 mm slice thickness, resulting in a voxel size of 0.01 mm3, 2.2 ms echo time, 17 ms repetition time, 2 averages, 15° flip angle.

The number of frames per cardiac cycle was determined by the heart rate (8 to 10 frames for 350–450 beats/min). End-diastolic (EDD) and end-systolic (ESD) diameters with the tips of the mitral valve leaflets in the middle of the sector were determined from MR frames analysis (OsiriX Imaging Software). Those diameters were used to assess the ventricular ejection fraction (EF): EF = (EDD-ESD)/EDDx100.

**Fig 1:**
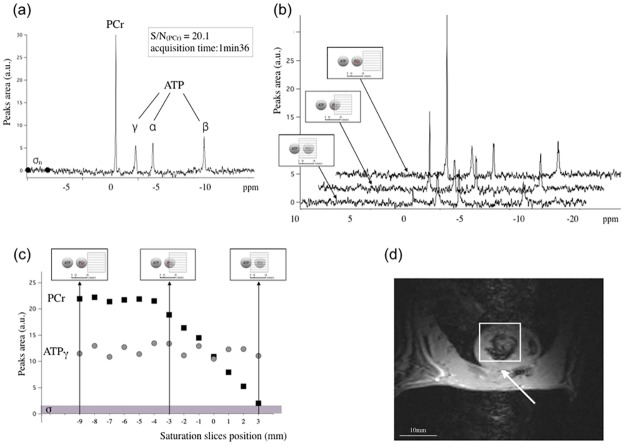
Results obtained on tubes containing PCr and ATP to calibrate the OVs spectroscopy method. a: a typical spectrum with signal/noise (S/N) ratio of 20 obtained with the OVS method in 1 min 36 s, with the saturation bands positioned outside of the tubes. b: spectra obtained for different positions of the saturation slice. c: PCr and ATPγ signal intensities as a function of the position of the saturation slice covering the tube containing the PCr. The reference in position is between the two tubes. The horizontal bar at the bottom of the graphs shows the noise level (σ_n_). d: Typical positioning of saturation slices (around the virtual square) in cardiac localized spectroscopy in vivo that takes into account the 3 mm overlap required to ensure the attenuation of ^31^PCr signal from (non cardiac) chest skeletal muscles.

Based on MR parameters determined by the prior optimizing procedure on the Eppendorf tube (see results), ^31^P spectra were obtained by the accumulation of 128 free induction decay signals acquired during 3min12 (200 μsec block pulse, 90° flip angle, 1.5 s repetition time, 8 kHz BW; 4096 data points). Localized spectra were obtained in a volume that visually delimited the left ventricle in diastole as determined by positioning the six saturation slices on 3D views of the heart (see Fig 1d).

### Follow-up experiments

MR-Imaging followed by the acquisition of localized ^31^P spectra were performed weekly at regular interval for each animal (same day of the week) from 12 to 21 weeks of age (10 weeks follow-up).

### Cardiac PCr/ATP response to IsoP injection

At 21 weeks of age, after MR-Imaging and the acquisition of two localized spectra in basal conditions, a bolus (0.15 ml) containing 10μg/kg body mass of isoproterenol diluted in 0.9% NaCl was injected i.v. into the tail previously equipped with a catheter. A series of spectra was then acquired as soon as tachycardia accounted for the physiological effect of IsoP, which usually took only a few seconds after injection. The first spectrum acquired in 3min12s after injection was used to quantify the magnitude of the immediate drop in cardiac PCr/ATP in response to IsoP. The following spectra were used to assess how the magnitude of the initial drop in PCr/ATP progressively vanished as a function of time. The time constant of the exponential fit of the decay in magnitude was used as an indicator of cardiac PCr/ATP recovery after isoproterenol injection.

### Total experimental time

Experiments during the follow-up included animal preparation (15 min), MRI analysis (20 min) and ^31^P MRS acquisition (15 min) and lasted ± 60 min. Some 30 additional minutes were needed the last week of experiment (age 21 weeks) to obtain the time course of PCr/ATP in response to ß-adrenergic stimulation.

### Statistical analyses

Quantitative measurements were expressed as mean ± standard error to the mean (SEM). *In vivo* MR studies were analyzed using repeated measures ANOVA followed by paired or unpaired *t-*test with Bonferroni corrections for multiple comparisons (Prism 6, Graphpad software). Unpaired Student’s test was used for other parameters. Results were considered significant at *P*<0.05.

## Results

### Localized spectroscopy

The spectrum acquired with the ISIS method of the Eppendorf tubes resulted in a S/N of 20 for the PCr peak (data not shown), with a 12 min 48s acquisition time. Identical S/N was obtained on the same peak with the OVS method in only 1 min 36 s (Fig 1a). Fig 1b–1d display the results of the OVS method with regard to spatial selectivity.

The influence of the (1 mm) step-by-step displacement of the saturation slice on the attenuation of the PCr signal is illustrated by selected spectra in Fig 1b. The intensity of the PCr signal attenuation as a function of the slice position (Fig 1c) showed that the full covering of the PCr-tube (position 0 in Fig 1c) did not result in total cancelling of the PCr signal. Regarding to noise level (indicated in Fig 1c), such the attenuation of the PCr signal was considered sufficient for quantification of the PCr/ATP ratio in vivo. Therefore, a gap of 3mm was systematically applied to avoid contamination of the metabolites of the chest skeletal muscles on the spectra acquired on the heart (Fig 1d).

### Physiological characteristics of the experimental animals

**Fig 2:**
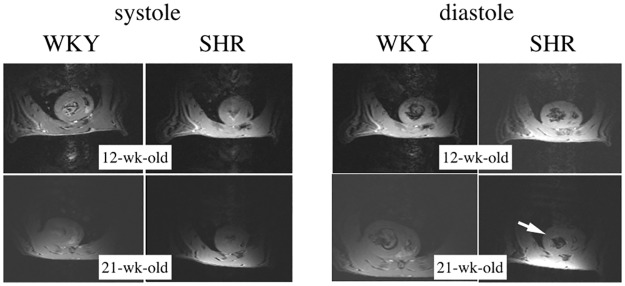
Typical short-axis cine-MRI frames obtained in vivo at 9.4T with the surface coil that allowed the assessment of the left the ventricle wall thickness. Typically, the frames obtained at the end of the ventricle relaxation (diastole) show the presence of concentric hypertrophy in hypertensive (SHR) rats (see the arrow) at age 21-wk while the phenomenon was absent at 12-wk in SHR as well at any age in control (WKY) rats.

At 10 weeks follow-up, a similar increase in mean body mass was observed in WKY and SHR (Table 1). Heart rate was slightly lower in SHR at onset of experiments and did not vary significantly thereafter (Table 1). ^1^H images were acquired with gated sequence to determine short axis view from which ejection fraction (EF) was calculated. Similar EF in SHR and WKY indicated the absence of abnormalities in ventricle functions at any time of the experiments. The main difference was on the thickness of the left ventricular wall that demonstrated the classic development of ventricle hypertrophy in the young SHR (Fig 2).

**Table 1:** Main characteristics in WKY and SHR at the beginning and at the end of the follow-up.

	Body weight (g)		Heart Rate (b.p.m.)		Ejection fraction (%)		[PCr] / [ATPγ]	
	12 weeks	21 weeks	12 weeks	21 weeks	12 weeks	21 weeks	12 weeks	21 weeks
WKY	350 ± 15	441 ± 16 ^#^	353 ± 29	303 ± 4	60 ± 2.1	57 ± 1.8	2.4 ± 0.1	2.2 ± 0.1
SHR	346 ± 12	432 ± 20 ^#^	311 ± 27 ^§^	312 ± 22	63 ± 1.3	61 ± 2.5	2.3 ± 0.1	2.4 ± 0.1

[PCr] indicates phosphocreatine concentration, [ATP] indicates adenosine triphosphate concentration. Ejection fraction was measured using cardiac magnetic resonance imaging and analysis by using OsiriX Imaging Software. Cardiac high-energy phosphates were determined using cardiac ^31^P spectroscopy. Note that the cardiac PCr/ATPγ ratio did not change.

Data are presented as mean ± SD. Changes at 21 weeks and 12 weeks and changes between WKY and SHR hearts were analyzed by Kruskal-Wallis test.

^#^ Statistically significant difference between 12 and 21 weeks old;

^§^ Statistically significant difference between WKY and SHR.

### Follow-up of cardiac PCr/ATP in vivo

**Fig 3:**
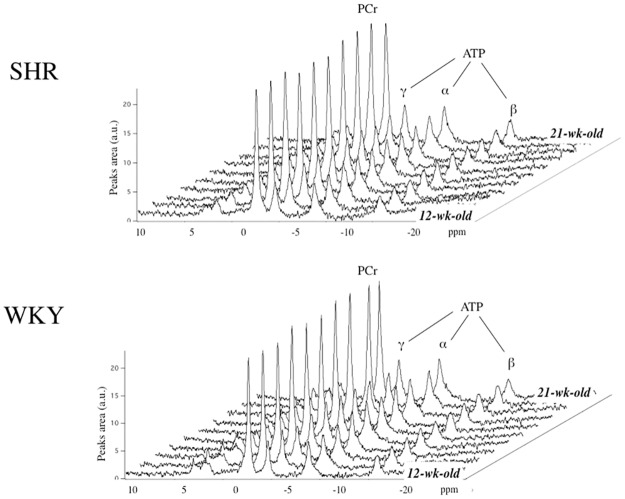
Typical cardiac localized 31P spectra obtained in vivo at 9.4T each week in control (WKY) and hypertensive (SHR) rats between the age 12-wk and 21-wk.

**Fig 4:**
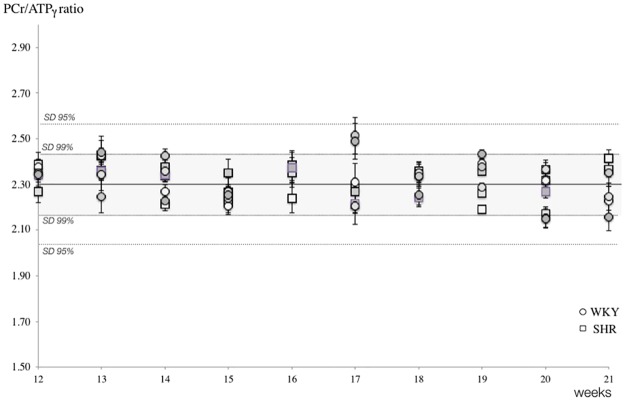
Weekly ratios of PCr/ATPγ in control (WKY, square symbols) and hypertensive (SHR, circles) rats. Horizontal lines indicate average values, 0.99 and 0.95 confidence intervals to highlight narrow intra- and owner-individual variations, great consistency in repeated ^31^P-MR assessments, and energy homeostasis along weeks.

Without repositioning the rat, typical localized cardiac spectra were acquired *in vivo* in basal conditions in WKY and SHR as shown in Fig 3. Spectra demonstrated high signal to noise ratio as well as high steadiness in peak areas along weeks. No significant differences appeared in PCr/ATP ratio in SHR compared to WKY neither at the onset of experiments nor at week 21 (Table 1**)**. Individual fluctuations in the cardiac PCr/ATP ratio are illustrated in Fig 4, in which the limit of confidence intervals 0.99 and 0.95 was added to illustrate the high reliability. Between these two time points and thanks to the capacity of NMR to follow-up individual cardiac PCr/ATP ratio in vivo, we demonstrated the absence of fluctuations in the myocardium energy status.

### Acute response to IsoP injection

**Fig 5:**
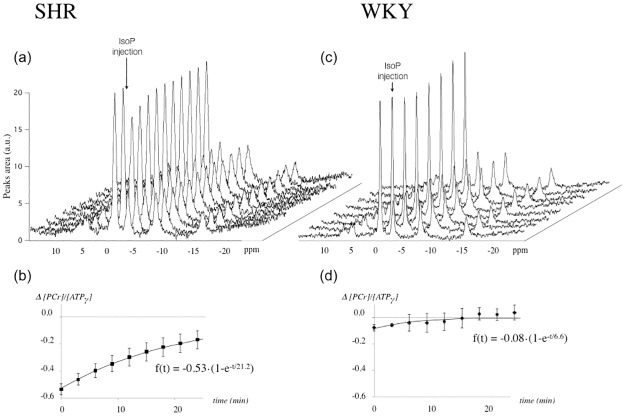
Typical cardiac localized ^31^P spectra obtained in vivo at 9.4T each 3min12 s before (two spectra in front) and immediately after the injection of isoproterenol (10μg/kg, indicated by an arrow) (a and b). Calculated areas under PCr and ATPγ peaks in every animals made it possible to plot the recovery of the PCr/ATP ratio after injection, fitted to a mono-exponential mathematical model (c and d). A significant slower rate of PCr recovery after the initial drop was found in SHR (c) compared to WKY (d) as specified by different time constants (respectively 21.2 for SHR and 6.6 for WKY, p<0.001).

Typical spectra obtained during the ß-adrenergic stimulation of the heart by a bolus injection of IsoP in a vein of the tail are shown in Fig 5. A small but significant drop in PCr/ATP was shown in WKY after IsoP injection, -3.5% ± 1.4. The main finding here was the more marked drop in SHR (-17.1% ± 0.8, P< 0.001). In each group, the drop in PCr/ATP lasted several minutes after injection as quantified by successive spectra during recovery (Fig 5). The cardiac PCr/ATP recovery was slower in SHR when compared to WKY as indicated by time constants of the exponential fit of PCr/ATP as a function of time (21.2 ± 6.1 min in SHR *vs*. 6.6 ± 2.5 in WKY, P<0.001).

Within the same period, we noted no differences in the time course of heart rate between SHR and WKY. Immediately after IsoP injection, HR increased by 35–40% in SHR (+125±25 bpm) as well as in WKY (+107±8 bpm). While tachycardia was sustained for several minutes, HR recovered after the initial increase with similar time courses in SHR and in WKY as assessed by time constants of exponential fits of HR as a function of time (11.2±5 min and 10.7±3.5 min respectively, *ns*.).

To resume, the combination of a greater drop in PCr/ATP associated with a lower rate of PCr/ATP recovery despite similar HR responses was the hallmark of the SHR heart *in vivo* in response to injection of the ß-adrenoreceptor agonist IsoP.

## Discussion

Here we report an original *in vivo* follow-up study on the course of cardiac energy homeostasis as heart disease develops in the young SHR. The prerequisite for a reliable analysis of cardiac energy regulation *in vivo* is the optimization of MR spectroscopy for the detection of small variations in the myocardial PCr/ATP-ratio that result from energy supply-demand imbalance. Most of cardiac spectroscopy studies rely on ISIS acquisition to obtain localized ^31^P spectra. However, the routine usually requires long acquisition time that may not be compatible with transient measurements of PCr/ATP changes. The OVS method used here was optimized (Fig 1) to obtain similar S/N in a 8 times shorter acquisition time, which was mandatory to characterize energy dynamics in response to ß-stimulation of the heart in vivo (Fig 5). In addition, shorter MR procedures are beneficial in reducing the total exposition time to anesthesia in the longitudinal study of diseased animals. Our pre-experiments for optimized spatial localization have shown that PCr/ATP measures are largely uncontaminated by skeletal muscle as far as saturation slices slightly overlap the region of interest. As a matter of fact, the MR procedure used in this study provided high-quality spectra in vivo (Figs 3 and 5) and values of myocardial PCr/ATP ratio (Table 1) were found within the range of those previously reported [20,24–29].

We investigated myocardial energy homeostasis *in vivo* by considering distinctively the follow-up of the PCr/ATP ratio as the disease develops (age 12 to 21 weeks) and the response to acute ß-adrenergic stimulation at a turning point (age 21 weeks) of cardiac energy remodeling in the young SHR [22].

### Follow-up of the myocardial PCr/ATP-ratio

One important finding in the present study was the consistency of *in vivo* cardiac MR spectra obtained in young SHR and WKY (Fig 3), which greatly improves our capacity to distinguish SHR data from control data. Weekly intra-individual variations in myocardial PCr/ATP were less than 2.18% ± 0.07% (Fig 4), which makes it possible to conclude with reasonable confidence that the myocardial PCr/ATP-ratio did not vary over time (age 12 to 21-wk), neither in WKY nor in SHR. This constitutes the first demonstration *in vivo* that the myocardial energy balance is unaltered in the young SHR during the sustained phase of hypertension as left ventricle hypertrophy progressively develops (Fig 2).

The perfect stability in myocardial PCr/ATP ratio in the SHR heart along weeks results from the permanent functioning of the supply-demand system, which has been quantitatively described by our applications of modular control analysis (MoCA) on perfused beating hearts [9]. MoCA teaches us that the steady-state of the PCr/ATP-ratio results necessarily from parallel activation of supply and demand rates, a regulation driven by calcium-acting hormones [9,12], a great responsiveness (elasticity in MoCA terms) of mitochondrial energy supply to small fluctuations in PCr, ATP and other connected energetic intermediates (ADP, Pi…), or both [11]. As regards responsiveness, it has been shown in isolated myocytes obtained from the young SHR heart that the ability of mitochondrial ATP synthase to respond to increased energy demand has disappeared, probably due to calcium abnormalities [30]. The quantitative relevance of this defect under *in vivo* conditions is still unknown to date. The intact PCr/ATP-ratio in basal conditions among SHR and WKY at any age in the present study indicates poor relevance of this mitochondrial defect. This could be the consequence of concomitant balancing mechanisms involved in mitochondrial energy export, *e*.*g*. an overexpression of the adenine nucleotide translocator (ANT) as demonstrated in cardiomyocytes of the SHR heart [31]. ANT facilitates ADP/ATP exchange across the mitochondrial membrane, which impart to this protein a central role in the integrated mitochondrial responsiveness to ATP demand [32]. It has been shown that ANT function change according to developmental or pathological state of the heart [33,34]. Additional mechanistic studies combining *ex vivo* and *in vivo* investigations are needed to assess the specific adaptation of maturation-dependent mitochondrial responsiveness in the young SHR heart.

Parallel activation seems intact and healthy in the young SHR heart in basal conditions, which allows a perfect balance between energy supply and energy demand rates. It may seem a paradoxical finding that energy balance is preserved despite alterations in calcium dynamics reported in the young SHR heart [16,17], unless calcium disturbances were progressive enough to allow the development of adaptive processes. The study of heart in basal conditions says yet nothing on the ‘reserve’ of parallel activation and the true efficiency of the adaptation to calcium defects, an issue which requires to test the heart response to calcium-mediated ß-adrenergic stress.

### Acute regulation by ß-adrenergic stimulation

The high point of the present study was the illustration of an abnormal cardiac energy regulation by acute stress of energy metabolism in the SHR heart *in vivo*. Thanks to successive *in vivo* MR acquisitions and reasonable acquisition time, we were able to assess kinetically the response of the myocardial PCr/ATP-ratio to a bolus of isoproterenol injected in a vein of the rat tail. Despite similar time course of heart rate (tachycardia) among SHR and WKY, the SHR heart demonstrated a higher drop in myocardial PCr/ATP followed by a slower recovery rate after isoproterenol injection (Fig 5). Both the marked drop and the slow recovery indicate a blunted response of ATP supply relatively to an increased ATP demand. This is a sign of failure in parallel activation of ATP supply and demand, which could originate from an altered sensitivity at ß-receptors level and/or altered calcium-mediated signal. It also indicates the incapacity of mitochondrial responsiveness to ATP demand to rescue the failing parallel activation in the whole homeostatic regulation *in vivo*. We previously demonstrated in the isolated perfused heart that the ß-adrenergic stimulation of the heart is a reliable way to evaluate parallel activation in the beating heart [12]. The circulating catecholamines, noradrenaline and adrenaline, exert their inotropic effects by activation of multiple adrenoreceptors including ß_1_-, ß_2_- and ß_3_-adrenoreceptors as does isoproterenol, a potent agonist of all three types of ß-adrenoreceptors [35]. The inotropic effect of ß-stimulation at myocytes level comprises a complex physiological mechanism involving marked modulations in both mean calcium cytosolic concentration and rises in calcium transients [36,37]. During the early phase of disease in the SHR heart prolonged action potential and increased excitation-contraction coupling gain occurs and synergistically increase Ca^2+^ transients [16,17]. Das et al. [30] showed that a role of mitochondrial Ca^++^ is implicated in the abnormal mitochondrial ATP synthesis response in the SHR. While the phenomenon could be of poor relevance in basal conditions as discussed above, the ß-adrenergic test here shows that it likely plays a critical role in the defect of cardiac energy homeostasis in the stressed heart.

### Study limitations

The present study suffers from a number of limitations. Here under β-adrenergic stimulation, our prime aim was the dynamic acquisition of ^31^P spectra to assess the recovery period for both groups of rats. As a consequence, the assessment of ventricular ejection fraction, which requires to run an additional time consuming cine-MR images procedure could not be evaluated in the time course of ß-adrenergic stimulation. As well, although heart rate has been reliably assessed during NMR experiment and demonstrated similar responses in both groups, no detailed ECG recordings were available due to instrumental constraints (no electrode on chest to avoid magnetic field perturbation) and altered electrical signals (QRS complex) in presence of high magnetic field.

Although a difference in ATP demand/supply matching was noted here between SHR and control rats, better insight could be provided by investigating creatine kinase flux as assessed by saturation transfer experiments [38–40]. This method has been widely used on ex vivo perfused hearts [38] and recently in exposed heart (open-chest) of swine with the probe sutured on the epicardium [39,41]. Although current research substantially improve saturation transfer method for a better understanding of the role of enzyme kinetics in ventricular function, its applicability to cardiac spectroscopy in vivo in the intact rat remains limited [42].

In conclusion, the present study demonstrates that in vivo cardiac ^31^P MR spectroscopy at 9.4T provides spectra of sufficient quality for reliable systems-level analysis of myocardial energy homeostasis in the intact rat. As a number of heart disease models exists in the rat, such a non-invasive method is obviously helpful to investigate the course of myocardial energetics as heart disease develops. Furthermore, thanks to reasonably short data acquisition time, the localization using saturation slices *in vivo* adds significant value for studying transient myocardial energetic responses to sudden stress.
